# Urate in fingernail represents the deposition of urate burden in gout patients

**DOI:** 10.1038/s41598-020-72505-6

**Published:** 2020-09-23

**Authors:** Haibing Chen, Lili Zhao, Fengjing Liu, Si Chen, Zhumeng Hu, Lihui Chen, Yiwen Ma, Kaifeng Guo, Aichang Ji, Tony R. Merriman, Jun Zhe Min

**Affiliations:** 1 Department of Endocrinology and Metabolism, Shanghai Eighth People’s Hospital, Shanghai, 200233 China; 2 Department of Endocrinology and Metabolism, Shanghai 10th People’s Hospital, Tongji University, Shanghai, 200072 China; 3 Key Laboratory of Natural Medicines of the Changbai Mountain, Ministry of Education, Pharmaceutical Analysis, College of Pharmacy, Yanbian University, Yanji, 133002 Jilin Province China; 4 Department of Endocrinology and Metabolism, Shanghai Jiaotong University Affiliated Sixth People’s Hospital, Shanghai, 200233 China; 5 Shandong Provincial key Laboratory of Metabolic Diseases, the Affiliated Hospital of Qingdao University, Qingdao, 266555 China; 6 Department of Biochemistry, University of Otago, Dunedin, New Zealand

**Keywords:** Biomarkers, Medical research, Rheumatology

## Abstract

Urate in the fingernails of gout patients and healthy volunteers was successfully detected by high-performance liquid chromatography (HPLC) with ultraviolet (UV) in our previous research. This study aimed to further investigate whether nail urate could be a proxy for the burden of monosodium urate (MSU) crystals deposits in gout. To this end, we conducted a study in two parts. Firstly, we successfully detected urate in the nail by HPLC–UV and evaluated nail urate concentrations in control subjects and patients with gout. As expected, we found that levels of nail urate were significantly higher in patients with gout than in healthy controls, and the nail urate level was significantly correlated with the volume of MSU crystals deposits measured by dual-energy CT (DECT). Secondly, we found that nail urate can reflect changes in urate levels in the body during urate lowering therapy through a 3-month follow-up study. Our results provide the possibility of quantification of urate in human fingernails as a non-invasive alternative for assessing MSU crystals deposits in gout.

## Introduction

Urate is the end product of an exogenous pool of purines and endogenous purine metabolism^1^. Elevated serum urate levels (hyperuricemia) cause gout. Across the globe, both hyperuricemia and gout have increased in prevalence over the last few decades^2,3^. Many studies reveal that hyperuricemia is associated with many diseases, including diabetes mellitus^4^, diabetic kidney disease^5^, cardiovascular diseases^6,7^, stroke^8^, hypertension^9^, dyslipidemia^10^. Deposition of monosodium urate (MSU) crystals in the joint spaces and tissues is the fundamental cause of gout. Long-lasting hyperuricaemia causes deposition of MSU crystals in the joints and soft tissues, triggering gouty arthritis and, if not properly treated, the formation of gouty tophi^11^.

Early diagnosis and treatment are essential for patients with gout. At present, the diagnosis of gout is mostly based on the classification criteria established by The American College of rheumatology (ACR) in 1977^12^. Although serum urate levels are recognized as the most important risk factor for gout, not all patients with hyperuricemia will develop gout^13^. In addition, during the acute attack of gout, serum uric acid levels may even be normal^14–16^. Moreover, the measurement of serum urate cannot assess the accumulation of urate deposits. In recent years, advanced imaging technology such as dual-energy CT (DECT) and ultrasound (US) have played an important role in the diagnosis of gout, which is reflected in the 2015 classification criteria for gout formulated by The American College of Rheumatology (ACR)^17^. These methods can reveal MSU crystals deposit**s** to assess urate burden in gout. However, these techniques are often expensive and not readily available, thus leaving the clinician reliant on the patient history and presentation. Therefore, to accurately assess the seriousness of the disease, and initiate appropriate therapy, it is desirable to have a simple and reliable detection method of urate burden.

In our previous research, we successfully detected urate in the fingernails of gout patients and healthy volunteers by high-performance liquid chromatography (HPLC) with ultraviolet (UV)^18^. Here, we aimed to investigate the correlation between nail urate and the volume of urate deposits measured by DECT and to explore the possibilities of quantification of urate in human fingernails as a non-invasive alternative for assess MSU crystals deposits in gout. In addition, through a 3-month prospective observation study, we aimed to observe the effect of urate -lowering drugs on changes of nail urate levels.

## Methods

### Study population

The male participants with gout (n = 82) and male healthy volunteers (n = 24) were recruited from the out-patient clinic at the Shanghai Clinical Centre for Diabetes (Shanghai, China). The diagnosis of gout was performed according to the 2015 Gout Classification Criteria^17^. We excluded people who had fungal infections or other diseases that affect the normal state of the nail. Patients receiving regular urate-lowering therapy for more than 2 weeks were also excluded.

Subjects with gout with febuxostat treatment (n = 25) or without febuxostat treatment (n = 9), and healthy volunteers (n = 4) were recruited from the out-patient clinic at the Shanghai Clinical Centre for Diabetes (Shanghai, China) for a 3-month follow-up study. Patients with a history of anti-hyperuricemic drug use, acute kidney injury, malignant tumor, blood system diseases, application of glucocorticoid hormone and immunosuppressant therapy and hepatic impairment were excluded. Patients with gout were treated with febuxostat at a dose of 40 mg/day for 3 months. Blood urate levels were measured at 0 weeks, 2 weeks, 4 weeks, 8 weeks, 10 weeks, and 12 weeks, and nails were collected for nail urate detection.

The study was conducted in accordance with the Declaration of Helsinki and approved by the Ethics Committee of Shanghai Jiao Tong University Affiliated Sixth People’s Hospital. All study participants provided written informed consent prior to enrolment.

### Anthropometric and biochemical measurements

Demographic and clinical data, including age, sex, duration of gout, weight and height were recorded. BMI was calculated as weight in kilograms divided by squared height in meters (kg/m^2^). The laboratory measurements used in this study have been described previously^19^. Venous blood samples were collected after an overnight fast for measurements of triglyceride (TG), total cholesterol (TC), low-density lipoprotein cholesterol (LDL-C), high-density lipoprotein cholesterol (HDL-C), fasting plasma glucose (FPG), glycosylated haemoglobin (HbA1c), serum urea nitrogen, serum creatinine and serum urate on a Hitachi 7600 analyzer using an enzymatic assay (Hitachi, Inc., Tokyo, Japan).

### Determination of urate in fingernail

We used the methods reported in our previous study to collect human nail samples and to determine urate in human fingernail^18^. In this study, the nails exposed to the edge of the finger of the second, third and fourth fingers of the non-dominant arm were chosen for specimen collection, and mixed them together for further processing. Nail samples were collected only once for each person. Briefly, the human fingernail samples were rinsed with 1.0 mL of 0.1% sodium dodecyl sulfate (SDS) for 1.0 min by ultrasonication. The procedure was repeated another two times. After rinsing, SDS was removed by three washings with distilled water. The fingernails were then dried in a desiccator under reduced pressure. The dried finger nails were crushed into a powder using a Shakeman 3 (BioMedical Science, Tokyo, Japan). A crushed fingernail sample (5.0 mg) was weighed into a 2.0 mL polypropylene tube. One hundred microliters of a 0.1 M NaOH solution was added. The mixture was kept at 90 °C for 20 min to extract the urate, then vortex-mixed for 30 s and centrifuged at 3,000×*g* for 1.0 min, and repeated once. All the supernatant fluids was collected and 100 μL of an internal standard solution (100 μg/mL hypoxanthine) was added. The resulting residues were added to 20 μL of 15% HCl for neutralization. Each 10 μL portion of the neutralization mixture was then subjected to HPLC–UV analysis.

### DECT examination

All subjects underwent DECT and the volume of MSU crystals was determined as previously described^15^. Briefly, DECT scans of the knees and feet were performed using two X-ray tubes and corresponding detectors (SomatomForce; Siemens Healthcare, Forchheim, Germany). In DE mode, these tubes scan at two different kilovolt levels, with simultaneous acquisition of two datasets, allowing for material-specific differences in the attenuation of the scanned tissue. The scan parameters were as follows: detector collimation 2 × 128 × 0.6 mm and rotation time 500 ms. The kilovolt level of tube A was set to 80 kV and the level of tube B was set to sn150 kV. Tube current parameters for feet were as follows: tube A,125mAs; tube B, 83 mAs. Transverse sections were reconstructed from the DE datasets, with kernel of Br40 and Qr40. Sagittal and coronal reformations in bone window settings were performed. The transverse soft tissue kernel datasets of both tubes were loaded onto a SyngoVia Workplace (Siemens Healthcare, Forchheim, Germany) and reconstructed with a commercially available software program (CT DE Gout; Siemens Healthcare, Forchheim, Germany). The total volume of urate crystal deposits was automatically quantified in cubic centimetres by the software. If no tophus was visible, the DECT volume of the index tophus was defined as 0. This software uses automatic colour-coding visualization to detect MSU crystals. The colour-coding datasets were reconstructed and reviewed in the transverse, sagittal and coronal image planes, with a slice thickness of 2 mm and an increment of 0.6 mm. All scans were obtained without intravenous contrast agent.

### Statistical analysis

All statistical analyses were carried out using SPSS version 17.0 (SPSS, Chicago, IL, USA). Continuous variables were expressed as mean ± standard deviation, and compared by two-sided t-tests or, if the data were not distributed normally, variables were expressed as median with interquartile range and the Mann–Whitney U-test was used. Categorical variables were expressed as percentages and using a chi-squared test or Fisher’s exact test. Correlations among the study variables were tested by the Pearson’s correlation coefficient. Those parameters with *P-*value < 0.05 in the Pearson’s correlation analysis were subsequently entered into multiple linear regression analysis with nail urate as a dependent variable. A *P-*value < 0.05 (two-sided) was considered statistically significant.

## Results

### Clinical characteristics of control subjects and patients with gout

Baseline characteristics of 106 subjects are shown in Table 1. There was a statistically significant difference in age between the groups (*P* < 0.05). However, after controlling for age, patients with gout had significantly higher body mass index (BMI), fasting glucose (FPG), blood pressure, trigylcerides (TG), serum creatinine levels and a higher frequency of diabetes, hypertension and non-alcoholic fatty liver disease (NAFLD) than those without gout. Meanwhile, urea nitrogen (BUN), total cholesterol (TC), low-density lipoprotein cholesterol (LDL-c) and high-density lipoprotein cholesterol (HDL-c) did not differ between the groups.

**Table 1:** General and clinical parameters of control subjects and Gout.

	Control	Gout
Case (M)	(24)	(82)
Age (years)	44.5 ± 13.2	54.8 ± 26.7^#^
Duration of gout (years)	–	9.4 ± 6.5
SBP (mmHg)	120.3 ± 24.2	130.8 ± 25.9***
DBP (mmHg)	78.1 ± 11.4	86.2 ± 24.3***
BMI (kg/m^2^)	23.4 ± 3.1	26.0 ± 4.5***
FPG (mmol/l)	5.0 ± 0.5	5.5 ± 0.6***
TC (mmol/l)	5.0 ± 0.9	4.8 ± 1.4
LDL-c (mmol/l)	2.3 ± 1.0	2.7 ± 1.0
TG (mmol/l)	1.4 ± 0.9	2.9 ± 1.6***
HDL-c (mmol/l)	1.7 ± 0.3	1.2 ± 0.5
BUN (mmol/l)	4.9 ± 1.2	4.6 ± 1.5
Serum urate (µmol/l)	314.5 (250.8–356.8)	530.0 (482.5–588.8)***
Nail urate (ng/mg)	50.0 (32.9–82.9)	170.4 (120.0–570.3)***
Cr (µmol/l)	71.5 (55.0–77.5)	85.0 (71.0–95.0)*
Hypertension	1 (4.2%)	40 (48.2%)***
Diabetes	0	7 (8.4%)*
NAFLD	6 (25.0%)	46 (55.4%)***

Data are expressed as means ± SD. Data were analyzed using independent-sample *t* test and covariance analysis (adjusted for age).

*SBP* systolic blood pressure, *DBP* diastolic blood pressure, *BMI* Body Mass Index, *FPG* fasting plasma glucose, *TC* total cholesterol, *TG* triglycerides, *HDL-c* high density lipoprotein cholesterol, *LDL-c* low-density lipoprotein cholesterol, *BUN* Urea nitrogen, *Cr* Creatinine, *NAFLD* non-alcoholic fatty liver disease.

^#^*P* < 0.05 vs. control; *P < 0.05,**P < 0.01, ***P < 0.001 vs. control; adjusted for age.

### Comparison of fingernails and serum urate between control participants and gout

**Figure 1:**
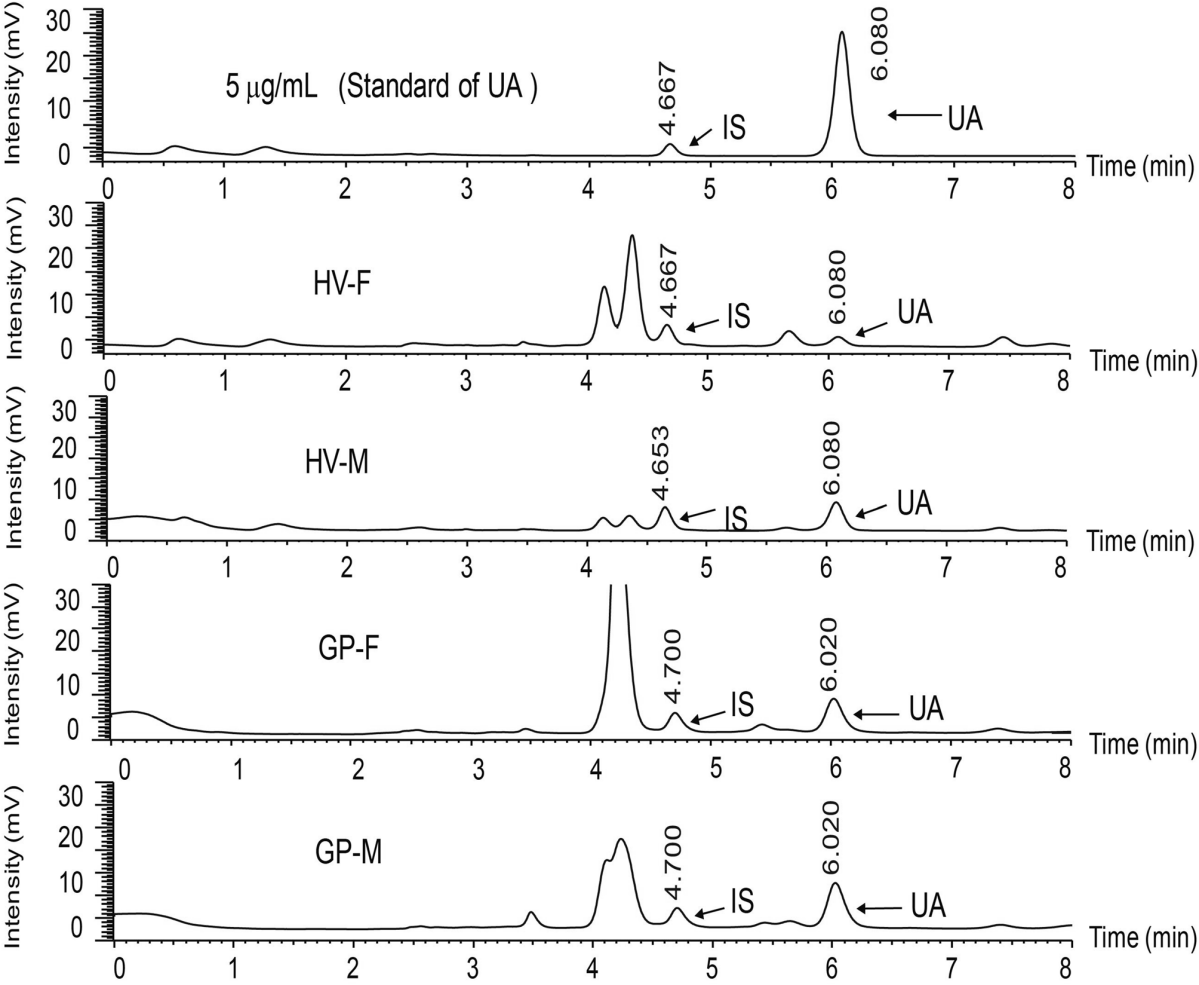
UV chromatograms obtained from urate (UA) in fingernails of healthy volunteer and gout patient by HPLC–UV.

We examined urate concentrations in fingernails and serum of all 106 study participants. Figure 1 shows the HPLC–UV chromatograms obtained from urate in the fingernails from the control subjects and gout. The peaks corresponding to the urate were completely separated without any interference from the endogenous substances in the human fingernails.

**Figure 2:**
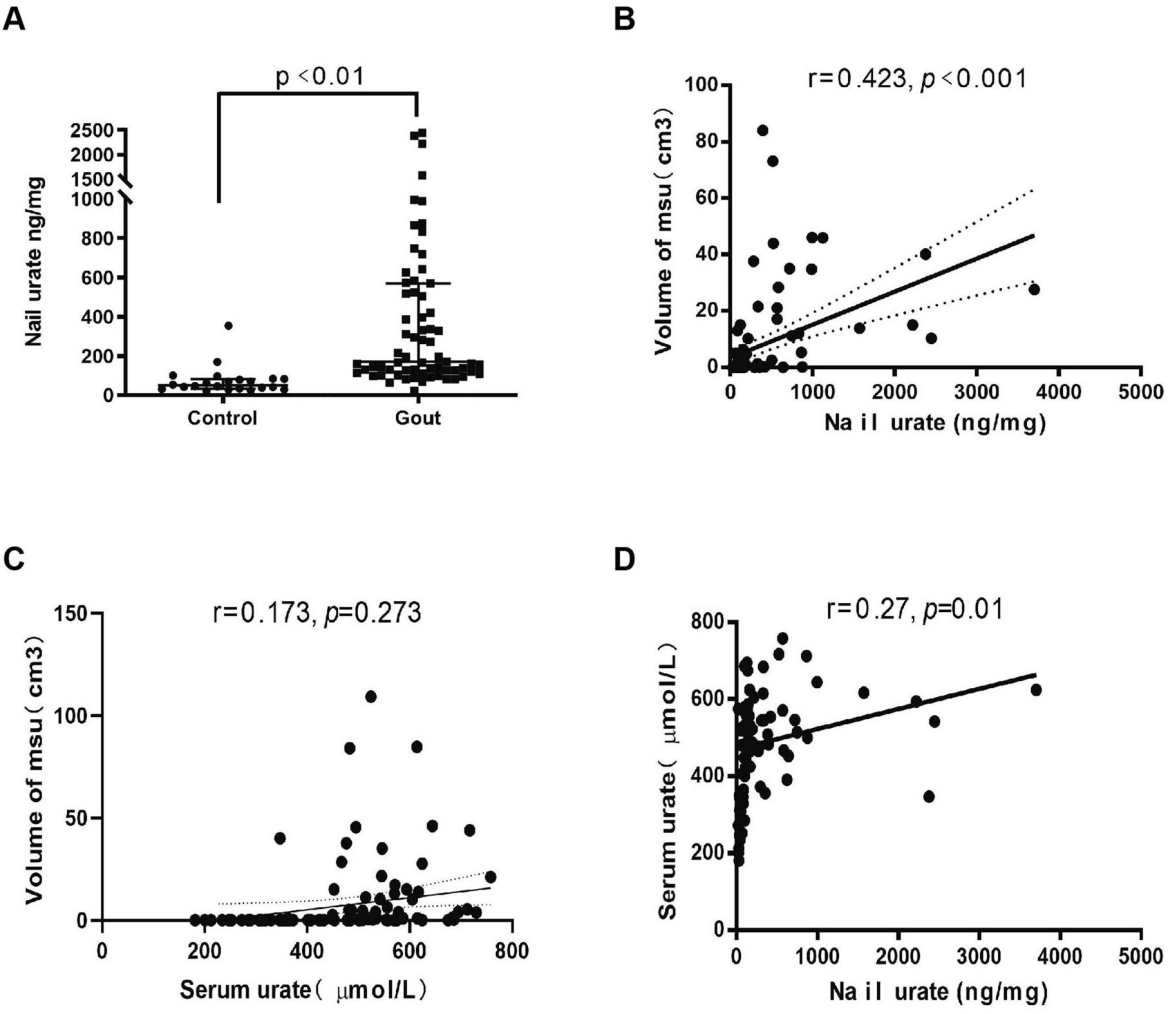
(**A**) Statistical analysis of nail urate concentration in the healthy volunteers (n = 24) and gout patients (n = 82); (**B**) correlation between urate concentrations in nail and the volume of MSU crystals measured by DECT in total 106 subjects; (**C**) correlation between urate concentrations in serum and the volume of MSU crystals measured by DECT in total 106 subjects; (**D**) correlation between urate concentrations in serum and nail in total 106 subjects.

Using this method, the amounts of urate in the fingernail of the control subjects and gout were determined. As expected, urate concentration in the serum was significantly higher in the gout group compared to the control group (*P* < 0.001, Table 1). Moreover, we found that levels of nail urate was also significantly higher in patients with gout (median 170.4 ng/mg; interquartile range: 120.0–570.3 ng/mg fingernails) than in healthy controls (median 50.0 ng/mg; interquartile range 32.9–82.9 ng/mg, *P* < 0.001, Fig. 2A). Next, we investigated the relationship between serum urate and nail urate. The analysis demonstrated a significant positive association between serum urate and nail urate (r = 0.27; *P* = 0.01, Fig. 2D). Although this correlation is weak, these results suggest that a significant linear relationship may exists between urate concentrations in serum and fingernails. They also suggest the possibility of estimating urate concentrations in serum from that in fingernails, and potentially replacing blood collection with non-invasive fingernail collection.

### Relationship between nail urate levels and volume of MSU crystals

We investigated the correlation between nail urate and the volume of MSU crystals measured by DECT by testing the relationship of nail urate with a cluster of anthropometric parameters and biochemical indices include the volume of MSU crystals measured by DECT (Table 2). Pearson’s correlation analysis showed that the nail urate was positively correlated with serum urate (r = 0.27, *P* = 0.01), the volume of MSU crystals (r = 0.42, *P* < 0.001) (Fig. 2B), duration of gout (r = 0.35, *P* = 0.011), SBP (r = 0.30, *P* = 0.005), DBP (r = 0.26, *P* = 0.014) and fasting plasma glucose (r = 0.27, *P* = 0.041), but not correlated with age (r =  − 0.03, *P* = 0.793) in all 106 subjects. The multiple linear stepwise regression analysis revealed that nail urate was independently correlated with the volume of MSU urate deposits (standardized β = 0.34; t = 2.34; *P* = 0.025). Furthermore, the duration of gout was another independently factor that correlated with the volume of MSU crystals deposits in the multiple linear stepwise regression analysis (standardized β = 0.33; t = 2.27; *P* = 0.029). However, there was no significant positive correlation between serum urate and the volume of MSU crystals deposits (r = 0.17, *P* = 0.273, Fig. 2C).

**Table 2:** Correlations of nail urate with other variables in total 106 subjects.

	r	*p*
Age	− 0.03	0.793
BMI	0.13	0.235
Duration of gout	0.35	0.011*
FPG	0.24	0.041*
SBP	0.30	0.005**
DBP	0.26	0.014*
ALT	0.13	0.230
AST	0.15	0.229
TC	− 0.07	0.570
TG	− 0.03	0.785
LDL-c	− 0.12	0.373
HDL-c	− 0.07	0.599
BUN	0.17	0.145
Volume of MSU crystals	0.42	< 0.001***
Serum urate	0.27	0.010*
Cr	0.02	0.853

Data were analyzed using Pearson’s correlation analysis.

*SBP* systolic blood pressure, *DBP* diastolic blood pressure, *BMI* Body Mass Index, *FPG* fasting plasma glucose, *ALT* alanine aminotransferase, *AST* aspartate aminotransferase; TC, total cholesterol, *TC* total cholesterol, *TG* triglycerides, *HDL-c* high density lipoprotein cholesterol, *LDL-c* low-density lipoprotein cholesterol, *BUN* Urea nitrogen, *Cr* Creatinine.

*P < 0.05, **P < 0.01, ***P < 0.001.

### Effect of febuxostat therapy on nail urate levels in people with and without gout

**Figure 3:**
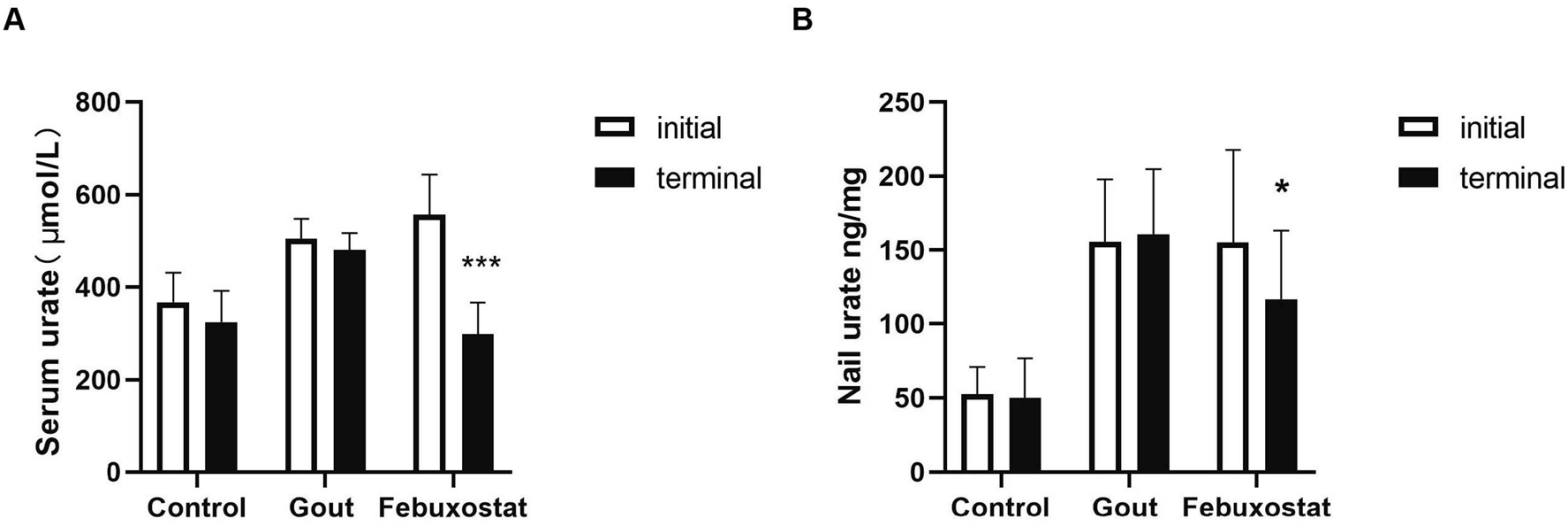
(**A**) Serum urate levels before and after febuxostat treatment for three months. (**B**) Nail urate levels before and after febuxostat treatment for three months. *P < 0.05, ***P < 0.001.

**Figure 4:**
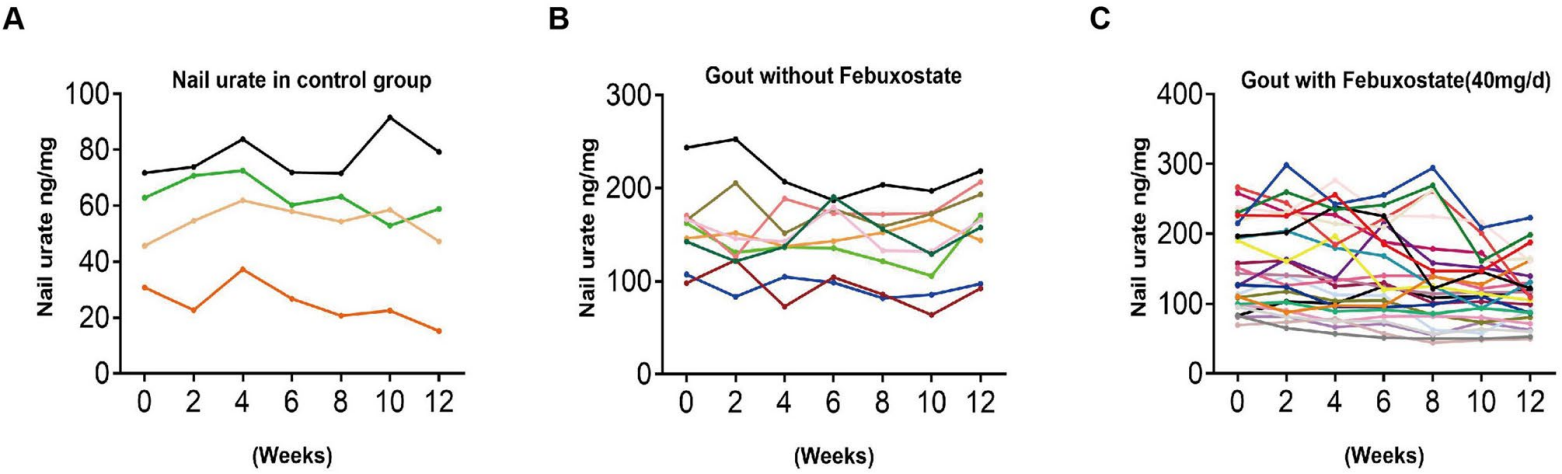
(**A**–**C**) the dynamic change of nail urate levels in control group (**A**), gout without febuxostat (**B**) and with febuxostat (**C**) for 3 months.

Supplementary Table S1 summarizes the anthropometric characteristics and metabolic variables at baseline. After 3 months of follow-up, patients with gout who received febuxostat had a significant decrease in serum urate levels compared with healthy controls and untreated group (*P* < 0.001, Fig. 3A). Moreover, we found that the nail urate level in the treatment group also decreased significantly (*P* < 0.05, Fig. 3B). As the treatment progressed, the nail urate gradually declines (Fig. 4C), but there is no obvious change in the healthy controls group (Fig. 4A) and the untreated group (Fig. 4B). These suggest that nail urate can also reflect changes in urate levels in the body during urate-lowering therapy.

## Discussion

The human fingernail may serve as a non-invasive alternative to estimate MSU crystals deposits in gout. In the present study, we successfully detected urate in the nail by HPLC–UV and evaluated nail urate concentrations in control subjects and patients with gout. As expected, we found that levels of nail urate were significantly higher in patients with gout than in healthy controls and we found a strong positive association between nail and serum urate. Moreover, we found that nail urate can reflect changes in urate levels in the body during urate lowering therapy. Importantly, the novelty of our study is that we demonstrated for the first time that the nail urate level was significantly correlated with the volume of MSU urate deposits measured by DECT. Our results provide the possibility of quantification of urate in human fingernails as a non-invasive alternative for assessing MSU crystals deposits in gout. Although identification of MSU crystals within a tophus sample or a joint aspirate on light microscopy is the gold standard for the diagnosis of gout, the diagnostic confirmation with imaging is becoming more attractive for clinicians and patients due to its noninvasiveness^17^. Both DECT and ultrasound can detect MSU crystal deposition, and have shown good diagnostic accuracy, especially useful in patients with longer duration of gout. However, these technologies still have certain shortcomings in terms of efficiency and cost, and there is still a need to explore simpler and more convenient detection methods to diagnose and assess the severity of gout^15,20^.

Human nails, as a non-invasive biological sample, have recently attracted attention. Compared with other biological samples, nail samples make up for the limitations of blood, urine, saliva and other samples due to its slow growth and can reflect the longer-term exposure of disease or post-treatment status. Recently, studies have reported that physiological information can be obtained through nails and may be used as a non-invasive diagnostic biological sample for chronic diseases^21–24^. Tirado-González et al. performed a nail biopsy on a person who identified the presence of a fungal infection and serendipitously discovered the presence of MSU urate crystals in the nail of a gout patient^25^. Li et al. used HPLC–UV to detect the amount of urate in the human fingernail in 26 healthy controls and 22 gout patients, and the nail urate concentration was found to be significant different between the two populations. The above results suggest that the presence of urate can be detected in the nail and there is a significant difference between gout and healthy controls. The content of urate in the nail may represent long-term average urate level and a proxy for level of MSU crystals deposited in the tissue during a certain period of time, which may be several months because fingernails grow about 3 mm/month or 0.1 mm/day; toenails about 1 mm/month, with complete replacement achieved in 6–9 months^26–28^. At present, the mechanism of urate deposition in nails is still unclear, and it has not been reported how long the nail urate level can reflect the serum urate level. Further research is needed in the future.

In this study, we found that the nail urate level was significantly correlated with volume of urate deposits measured by DECT.This result indicated higher nail urate was a predictive factor of the volume of MSU crystals. While hyperuricemia is a strong risk factor for gout, our study did not find a significant association between serum urate level and MSU crystals deposits measured by DECT, which indicated that measurement of serum urate does not assess the accumulation of urate deposits and therefore cannot estimate the urate burden. The result that the duration of gout was independently correlated with the volume of urate deposits measured by DECT further conformed the previous findings that the duration of gout and serum urate level were significantly associated with the urate crystal volume.

This study has some limitations. First, the small sample size, and there was also a bias for patients in whom aspiration was performed. Second, the analyses were limited to the knees and feet. This was because there has not been a general consensus so far on which joints should be examined to assess urate burden in gout and the knees and feet are known to have more prevalent MSU crystals deposits identified by DECT. Third, we measured the serum urate value only once, due to the fluctuation of serum urate, the average value of multiple measurements may be a better assessment of the relationship between serum urate and nail urate and the volume of MSU. Finally, compared with serum urate detection, the cost of HPLC–UV detection is relatively expensive, and in the future, better optimization is needed in order to be less expensive, convenient to operate, and suitable for routine analysis.

## Conclusions

In conclusion, we found in the present study that the nail urate concentration was positively correlated with the volume of MSU crystals as measured by DECT in males with gout and that nail urate can also reflect changes in urate levels in the body during urate-lowering therapy. At present, there is no good quantitative index of whole-body MSU crystal deposition. Although serum urate is an important indicator for the immediate change of urate level in the body, nail urate can reflect changes in urate deposition in the body during urate-lowering therapy and determination of nail urate may provide useful information for management of gout in situations where blood sample analysis and image technology are not possible. In future, a large, prospective, multicentre trial is needed to assess the performance of this biomarker and to demonstrate its effectiveness in clinical practice when compared to currently accepted methodologies.

## Supplementary information

Supplementary Information
