# Supplementary material for: Urate in fingernail represents the deposition of urate burden in gout patients
Source: Sci Rep. 2020 Sep 23;10:15575. doi: 10.1038/s41598-020-72505-6 (PMC7511301; doi:10.1038/s41598-020-72505-6)
Supplement: Supplementary file 1 — Supplementary Information [file 41598_2020_72505_MOESM1_ESM.docx]

**Supplementary table 1:** Baseline clinical characteristics and serum urate levels in Control individuals and GOUT subjects with and without Febuxostat

|  | **Control** | **Gout with** **Febuxostat** | **Gout without Febuxosta** |
| --- | --- | --- | --- |
| **Case (M/F)** | 4(4/0) | 25(25/0) | 9(9/0) |
| **Age (years)** | 38.0±5.1 | 49.2±12.7 | 43.6±15.7 |
| **Duration of Gout (years)** | ---- | 7.06±5.61 | 0.7±0.8 |
| **SBP(mmHg)** | 123.0±6.7 | 134.7±13.9 | 129.2±14.5 |
| **DBP(mmHg)** | 78.0±4.5 | 85.2±11.90 | 89.3±6.4 |
| **BMI(Kg/m2)** | 24.0±0.6 | 27.2±6.8 | 24.5±1.4 |
| **FPG(mmol/l)** | 5.1±0.5 | 5.4±0.5 | 5.4±0.6 |
| **TC(mmol/l)** | 4.9±0.8 | 4.9±0.8 | 5.2±0.9 |
| **LDL-c(mmol/l)** | 2.9±1.0 | 2.8±0.8 | 2.6±1.3 |
| **TG(mmol/l)** | 1.9±1.0 | 2.8±1.5 | 2.9±1.9 |
| **HDL-c(mmol/l)** | 1.3±0.2 | 1.2±0.4 | 1.2±0.4 |
| **BUN(mmol/l)** | 5.1±1.2 | 4.2±1.1 | 4.2±1.2 |
| **Serum urate (µmol/l)** | 389.0±74.8 | 531.4±71.3 | 511.1±65.4 |
| **Cr(µmol/l)** | 68.6±7.8 | 82.5±27.2 | 47.0±36.0 |
| **Hypertension** | 0(0%) | 12(48.0%) | 3(33.3%) |
| **Diabetes** | 0(0%) | 0(0%) | 1(11.1%) |
|  |  |  |  |

Abbreviations: SBP, systolic blood pressure; DBP, diastolic blood pressure; BMI, body mass index; FPG, fasting plasma glucose; TC, total cholesterol; TG: triglycerides; HDL-c, high density lipoprotein cholesterol; LDL-c, low-density lipoprotein cholesterol; BUN, Urea nitrogen; Cr, Creatinine.
